# Towards Lateral Flow Quantitative Assays: Detection Approaches

**DOI:** 10.3390/bios9030089

**Published:** 2019-07-17

**Authors:** Alexandr E. Urusov, Anatoly V. Zherdev, Boris B. Dzantiev

**Affiliations:** A.N. Bach Institute of Biochemistry, Research Center of Biotechnology of the Russian Academy of Sciences, Leninsky Prospect 33, Moscow 119071, Russia

**Keywords:** immunoassay, rapid tests, immunochromatography, portable detectors, optical detection

## Abstract

Point-of-care (POC) or bedside analysis is a global trend in modern diagnostics. Progress in POC testing has largely been provided by advanced manufacturing technology for lateral flow (immunochromatographic) test strips. They are widely used to rapidly and easily control a variety of biomarkers of infectious diseases and metabolic and functional disorders, as well as in consumer protection and environmental monitoring. However, traditional lateral flow tests rely on visual assessment and qualitative conclusion, which limit the objectivity and information output of the assays. Therefore, there is a need for approaches that retain the advantages of lateral flow assays and provide reliable quantitative information about the content of a target compound in a sample mixture. This review describes the main options for detecting, processing, and interpreting immunochromatographic analysis results. The possibilities of modern portable detectors that register colored, fluorescent, magnetic, and conductive labels are discussed. Prospects for further development in this direction are also examined.

## 1. Introduction

Effective therapy is only possible when a disease is diagnosed quickly and reliably. According to Roche Molecular Diagnostics, only 2% of total healthcare costs go towards in vitro diagnostics, while 60% of treatment decisions are made based on in vitro information [1]. In recent years, there has been a trend towards diagnosis at the place of treatment, or point of care (POC). This includes tests during the initial examination of an admitted patient, large-scale screenings, doctors’ house calls, and self-diagnostics. The global market for medical analytical systems is rapidly growing, and in 2017, it reached over US $18 billion [2]. Other important practical fields that require POC tests are consumer protection and environmental monitoring. POC test systems make analyses less costly and much faster compared with the methods used at stationery laboratories; they quickly provide information necessary for making further decisions.

Among POC test systems, tests based on the lateral flow technique (immunochromatography) are widely used for a broad range of significant tasks. The first immunochromatographic tests were introduced in the 1980s for pregnancy self-testing [3]. This is still the most well-known and widely used application of the lateral flow technique. Immunochromatography is a key component of the modern methods used to control psychoactive compound oncomarkers, acute infarction markers, allergens, and microorganisms that cause various diseases, as well as in serodiagnostics to detect antibodies of pathogens in the blood [4,5,6,7,8]. In total, there are over a thousand types of immunochromatographic test systems for qualitative control of various analytes, including medical tests and solutions for environmental monitoring, quality control of products and raw materials, and analyses of natural and manmade objects.

**Figure 1:**
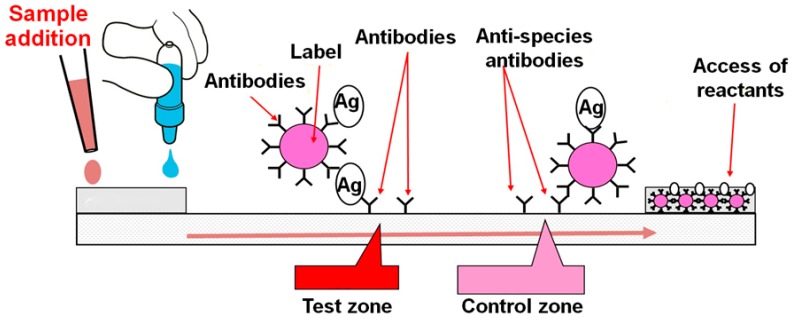
Common structure of lateral flow test strip and interaction of reactants in the course of the assay.

Immunochromatography is a well-adapted technique for POC diagnostics. In the classic version, all the reagents involved in the analysis are beforehand applied to the membrane components of a test strip. A test strip’s contact with a test sample causes a fluid to move along the membranes, which actuates specific interactions of immunoreagents and results in the staining of certain zones of the test strip (see Figure 1).

Traditionally, lateral flow tests were considered as tools for qualitative analyses, which involved the user’s conclusion that the controlled compound is present in the sample (or a certain threshold concentration of the controlled compound is exceeded) based on visual evaluation of the staining. The importance of the assay’s improvement and the quantitative output of POC testing follows from the possibility of obtaining concrete data on the level of a biomarker of a pathological process in medical diagnostics or a dangerous pollutant in environmental monitoring or consumer protection, thereby allowing more grounded decisions about protective measures. For example, the quantitative estimation of a level of inflammatory markers (C-reactive protein, procalcitonin, etc.) allows controlling the dynamics of therapeutic actions [9]. Another example is the control of stored food and feed products, where revealing toxic contaminants such as mycotoxins at levels lower than maximum residue levels makes it possible to start taking measures against fungal contamination before increasing levels of toxicants render the products unusable [10].

Over the past decade, a series of affordable and compact detecting devices for registration and quantification of immunochromatographic results has been designed [11,12,13]. The inclusion of instrumental registration in the immunochromatographic assay does not make it significantly more complicated. However, such detectors’ use is often limited to the storage of images and operator-independent (objective and automated) assessment of analysis results as “positive” or “negative”, without quantification of the analyte content. A new trend in this type of detection is the measurement of the analyte concentration in the tested samples. This paper reviews the modern tools for quantitative immunochromatography that are already available to users or that have good prospects for practical implementation in the near future.

## 2. Principles of Optical Signal Registration for Quantitative Lateral Flow Assays

**Figure 2:**
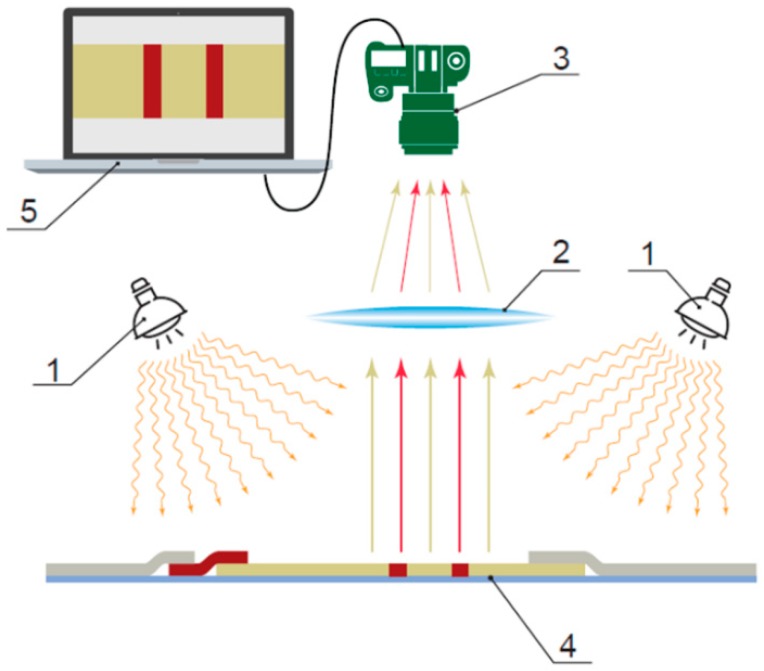
General principle of the optical registration of an immunochromatographic assay. Notations 1–5 are explained in the text.

The prevailing approach in lateral flow assays is optical registration of colored labels on the strip’s surface, the general principle of which is presented in Figure 2. The main elements of the measuring device are a light source(s) (1), a focusing system (2), a registering device (3) for the light reflected by a test surface (4), and instrumental and software tools for further processing the registered images (5).

The first developments in the 1990s focused on a construction that registered the total staining intensity (brightness of the reflected light) of certain areas of the test strip using a system of light-emitting diodes. In immunochromatography, the staining of a strictly defined zone of the test strip is significant, and thus the precise localization of staining is essential. For example, Blatt et al. developed a device with 28 light sensors along the test strip [14]. They demonstrated the possibility of measuring the content of *N*-telopeptide cross-linking domain of type I collagen (bone resorption marker) and creatinine in undiluted urine using colloidal gold as a label with minimal measured concentrations of these biomarkers equal to 30 nM and 1 mM, respectively, and very good stability coefficient of variation (CV) was (<0.1% over 270 s of registration) and reproducibility (CV ≤ 0.5%). The construction of such reflectometric devices has been significantly improved over time. Thus, the development of Askim and Suslick [15], published in 2015, provides a device sizing of 12.8 × 9.5 × 4.0 cm that can scan 28–48 colored spots printed at 1.2 mm center-to-center distances during 11 ms and gives a large amount of digital data with 2750 possible values of reflectance and an average standard deviation in the range of 1.95%–2.15%. However, due to the technological complexity of the formation of an array of detectors for high-resolution reflectometry, these technologies have never widely been used in diagnostic practice.

Portable digital cameras have become a convenient tool used to detect immunochromatography results because they easily and quickly produce full-color images of test strips [16,17]. Due to their mass manufacturing starting in the early 2000s, their cost has respectively decreased. Batch-manufactured cameras register images with a resolution of up to 2400 dpi, which corresponds to individual areas of less than 1 μm^2^ in size. Light sources with specified spectral features (often monochromatic) used in such detectors ensure high contrast between the background and the specific staining area.

Any digital optical detector represents an image of a test strip as data for an array of points arranged in horizontal and vertical rows. Each point, or pixel, is characterized by three numerical parameters corresponding to three channels of image formation—red, green, and blue (RGB)—comprising the RGB circuit. The registration results for each of the channels are integers that increase as staining intensity increases. In most detectors, this value ranges from 0 to 255. Information from the three channels can be integrated as grayscale image characteristics. However, separate color channels provide additional opportunities for processing the analysis results due to better separation of signals from bound labels and nonspecific matrix coloration.

Research conducted by Gui et al. [18] showed that while dealing with the R-channel data, the ratio of the specific and nonspecific signals increases more than three times compared with estimating a total (full-color) image. Using this approach, the authors reduced the limit of immunochromatographic detection of *Helicobacter pylori* antigens to 20 pg/mL with CdS quantum dots as fluorescent labels.

Which tools or methods to register images of test strips and transform them into quantitative results of lateral flow assays are available to users today?

## 3. Quantitative Assays with Optical Labels

### 3.1. Specialized Tools for Optical Detection

Specialized registering systems for lateral flow tests are produced by a number of manufacturers (Table 1). Their automatic work enhances the measurement objectivity (the operator is not involved in making a decision on the evaluation of the obtained results). Some of them (see BD Veritor System and Biohit Quick Test Reader as examples) provide only a qualitative conclusion after optical data processing, namely, confirmation of the visual reading of the test line as “not visible” or “visible”. However, the majority of these detectors can calculate the analyte content based on the line intensity and give this information as output of the measurements.

Most detector manufacturers supply their own test strips that are adapted to use in combination with such detectors. This package approach is vital to a correct quantitative analysis. When measuring for a random set of tests designed for qualitative analyses, a situation can occur in which the technologies used to manufacture some tests are somewhat different (due to special features of the manufacturers’ production lines, etc.). It does not affect the qualitative (“yes–no”) conclusion, but it may skew the data on the monitored analyte’s content.

It should be noted that most commercial detectors can be used only in combination with the test systems from the same manufacturer. Measurements of other test systems are limited by manufacturers via barcodes or other tools for labeling. If a manufacturer from the permitted list is not identified, the measurement process is terminated. These restrictions substantially limit the scope of quantitative immunochromatography use. In contrast, open-type detectors may be used in combination with lateral flow tests of different manufacturers and need only additional setup and calibration to give correct quantitative information about the analyte content using novel lateral flow tests. AXXIN AX-2X, Digital Strip Reader (Bio-AMD), ESE-Quant LFR, Immunochromato-Reader (Hamamatsu), Lateral Flow Tester LFT 100, FS-Scanner, opTrilyzer^®^Med, Scannex readers, Kinbio DL2032, and Reflecom Multitest are examples of such open-type detectors.

Recently, ultraportable hardware to analyze the results of lateral flow assays has entered the market. These systems include products such as the Digital Strip Reader from Bioamd and the INCLIX test strip cartridge with a built-in detector and display developed by Sugentech. An illustrative example is the Cube-Reader—a microreader by the Optricon company. This device has a cube shape, an edge length of approx. 41 mm, weighs 40 g, and combines all the features of optical registration for lateral flow test strips. The results are shown on a small display in a “yes–no” format, and all the controls and digital data processing results can be accessed through a smartphone or a computer via Wi-Fi or Bluetooth. Table A1 in the Appendix A summarizes the stated analytical parameters of some such quantitative test strips that are integrated with commercial detectors.

Optical registration allows implementing lateral flow assays that not only have colored labels but also fluorescent ones emitting at visible or infrared wavelengths [20,21]: organic low-molecular-weight compounds, quantum dots, and colored latex particles. When applying these tests, the analytical signal can be enhanced by the higher intensity of the exciting light. Also, choosing a combination of the label’s optical properties and the exciting light’s wavelength may reduce the background signal significantly. The corresponding decrease of the detection limit after changing colorimetric labels to fluorescent ones was found to be up to 10 times when detecting chloramphenicol [22,23] and diagnosing syphilis [24] and up to 5 times when detecting benzopyrene [25].

### 3.2. General Purpose Devices as Optical Detectors

Results of lateral flow assays can be registered not only by special detectors but also by mobile devices and office equipment [12,26,27,28]. There are examples of the successful use of mobile phones and smartphones for this purpose [29,30,31,32], including when dealing with fluorescent tags [33]. Examples of reached analytical parameters for these assays are collected in Table 2. Such detectors are convenient and easy to use, and they allow for performing analyses even in field conditions. In addition, the user does not need to buy any special devices.

Saisin et al. [42] recently indicated that a simple decision such as manually setting the exposure time (instead of automatic settings) for a smartphone camera (iPhone 5s) led to a fivefold improvement of the detection limit for photometric quantitative registration of the results of *Acidovorax avenae* subsp. *Citrulli* detection—10^5^ CFU/mL. In comparison with the visual readout, a 10-fold improvement was reached.

Analytical and biological applications for cell phones and smartphones have already surpassed macroscopic digital photography. Adding special lenses and accessories makes it possible to significantly expand their host of functions. Using smartphone cameras allows for quantitative detecting [43], which evaluates the intensity of binding zone coloration.

Among such solutions, the use of fluorescent detection is worth mentioning. Thus, Hou et al. [44] have developed a dual-modality imaging system based on a smartphone that is able to provide quantitative measurements for test strips with either colored or fluorescent labels. The device uses both white and ultraviolet lights, which can be chosen according to the type of used label (colored or fluorescent). Examples of detecting human chorionic gonadotropin (colored label) and carcinofetal antigens (fluorescent label) have demonstrated the low detection threshold, being equal to 0.037 μg/mL. Shah et al. [45] coupled ultraviolet-light-emitting diodes with long Stokes shift quantum dots to mobile phone fluorescence measurements without optical filters and reached a limit of detection for influenza A nucleoprotein up to 1.5 fmol.

Zangheri et al. [46] developed a portable analytic device that transforms a smartphone into a chemiluminescence detector for quantitative lateral flow analysis. The device consists of a smartphone with accessories that can be made using an inexpensive desktop 3D printer. The device has two parts: a cartridge with the strip and a smartphone adapter with a planoconvex lens connected to the camera and a cartridge-insertion slot. Once an operator has completed the analysis, both the smartphone and the cartridge are to be inserted into an assembled unit to make the measurement. To obtain the best imaging of the test strip, a built-in application for smartphone cameras and an autofocus system are used. The system provides quantitative detection of cortisol in saliva in the range of 0.3–60 ng/mL, which is adequate for a diagnostically significant range.

### 3.3. Software for Processing Images and Obtaining Quantitative Assay Results

Some manufacturers already supply special software for immunochromatography. Software installed in a device processes registered images and ensures quantitative analysis results are obtained. Some of these solutions include special software for Android, iOS, and Windows-based smartphones and tablets developed and offered by Novarum^TM^ Readers (novarumreader.com), SkanEasy (skannex.com), Mobile Diagnostic Rapid Test Reader (mobileassay.com), and so forth. This software ensures that prompt quantitative test results are obtained without any additional equipment. The products ensure a high level of security of data transmission and storage, and the platforms are easy to use and can save data on five devices and synchronize them with cloud data storage. Also, smartphones are now often used to control other reading devices, which allows for keeping the system portable and using it under near-patient conditions. Market leaders have moved towards solutions such as the abovementioned Cube-Reader by Optricon and a kit for multipoint analysis on test strips with a possibility of creating kinetic quantitative curves and diagrams in real time using Scienion (scienion.com). It should be noted that when dealing with test strip images made through traditional methods, the reproducibility of the results is often not very high. The reason for this is the impossibility of coordinating the camera’s and tests’ positions and ensuring steady and even lighting. It is not a technical challenge to fix a test strip’s position and to have it evenly lighted; however, it requires an individual adapter for each cell phone model [47].

An alternative approach to the digital imaging of test strips is using standard scanners. In this case, traditional scanning produces a high-resolution image of a test strip, and it can be processed using existing software; that is, no special solutions for immunochromatography are required because the analysis of a test strip image does not differ much from electrophoresis data processing, for which software already exists.

**Figure 3:**
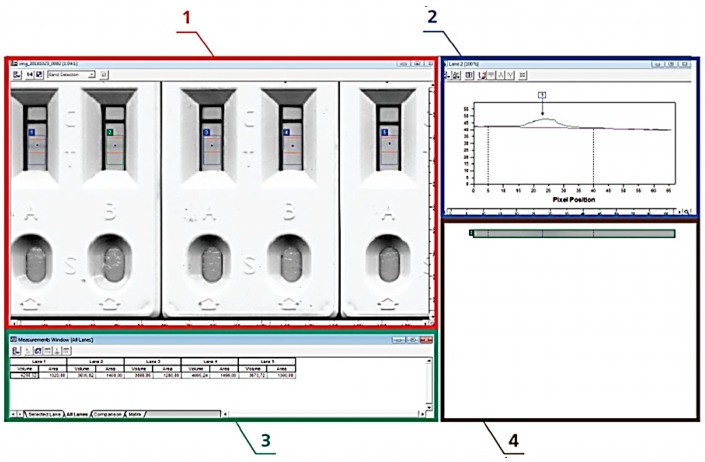
Working window in the Total Lab Quant software and its use for the quantitative assessment of immunochromatography results. (1) Image of test strips array, (2) distribution of staining intensity along the analyzed test strip, (3) identification data of the bonding zone and assessment of its staining intensity, and (4) zoomed-in image of the analyzed test strip.

Of the array of software accessible to users, the CLIQS 1D (TotalLab, totallab.com) is recommended, which ensures automated quantitative analysis of images and is user-friendly (Figure 3). MAIIA Diagnostics (MAIIA AB, maiiadiagnostics.com) has similar features, and it can work with the 16-bit—from 0 to 65,535—range of intensity values (shades of grey). Within 5–10 min, it produces data for 200–300 test strips. The most compact software for images analysis is the Gel Analyzer 2010a (gelanalyzer.com). Because it is only 250 Kb in size, it is capable of performing all necessary measurements on immunochromatography test strips.

## 4. Quantitative Assays with Magnetic Labels

**Figure 4:**
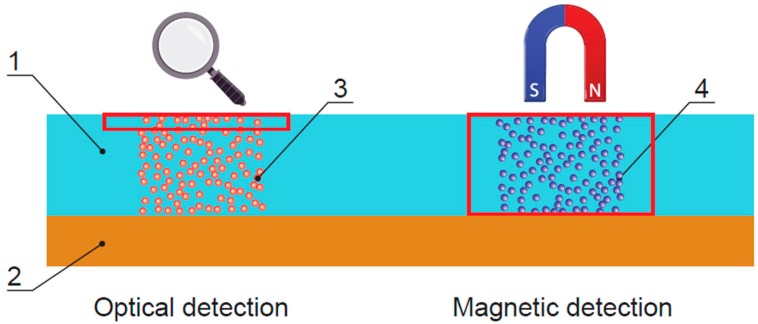
Comparison of optical and magnetic detection of immunochromatography results. (1) Contacting membrane, (2) plastic base layer, (3) colloidal gold or latex particles, and (4) magnetic particles. The membrane’s zones where the bonded labels are registered are highlighted in red.

For immunochromatography, magnetic particles can be used as labels. These particles are barely visible but are easily detectable due to their magnetic fields. The materials of test strips are not barriers to magnetic fields [48]; therefore, detection is possible not only in the upper layer (approx. 10 μm), as is in the case of colored particles, but in the entire depth (the strip is approx. 150–200 μm thick), which makes the assay features potentially better (Figure 4). The results of magnetic particle registering are not affected by the colored components of a sample, which in optical immunochromatography systems often results in substantial and uneven background coloring.

The effectiveness of magnetic detecting in immunochromatography has been proved by several targets. Thus, Shi et al. demonstrated immunochromatographic detection of the shellfish major allergen tropomyosin in a range of concentrations from 12.4 ng/mL to 20 μg/mL (3 orders of magnitude) [49]. The fish major allergen parvalbumin was detected in the work of Zheng et al. in a range of concentrations from 0.046 to 100 μg/mL. Lateral flow immunoassay for cardiac troponin I described by Xu et al. [50] had a sensitivity of 0.01 ng/mL with a detection range of 5 orders of magnitude. Shi et al. [51] demonstrated lateral flow immunoassay of *Listeria monocytogenes* with a range of measurements between 10^4^ and 10^8^ CFU/mL. Some companies have applied this method for the serial production of detectors (Table 3) in combination with specialized test systems.

## 5. Quantitative Assays with Electrically Conductive Labels

Interaction registration test strips may contain electrically conductive labels (usually metal nanoparticles) or excitant labels for the generation/transformation of conductive compounds (oxidizing enzymes). To take a measurement, a test strip’s specific interaction zone should be put against an electrode, most often made of inert substances (gold, platinum, and carbon). Registered parameters could be fluctuations of current, voltage, or resistance, and such analytical systems are respectively referred to as amperometric, potentiometric, or conductometric systems.

Currently, there are few examples of applications of this type of analysis compared with immunochromatography. Thus, Lu at al. described the detection of human chorionic gonadotropin by registering metal ionic labels [52]. Bismuth ions have been coupled with antibodies through a bifunctional chelating agent and released and quantified by anodic stripping voltammetry. By doing this, up to 1 mIU of gonadotropin can be detected.

Fernández-Sánchez et al. found the registering impedance of an electrochemical sensor coated with a pH-sensitive polymer within a prostate-specific antigen detection system with a limit of detection 3 ng/mL [53]. Lin et al. used cadmium-printed electrodes for the registration of the same compound in which cadmium is released as quantum dots that are dissolved [54]. They reached 2 orders of amplitude improvement in the detection limit (0.02 ng/mL) with high reproducibility of repeated measurements (the relative standard deviation was 6.4%).

Conductive labels, such as magnetic labels, can be registered throughout the entire depth of the test strip. Assay results are not affected by colored components of a tested sample but are affected by conductive components. The main factor that limits the practical use of systems that register the fluctuations of electric parameters on test strips is their insufficient sensitivity and reproducibility due to signal drift and matrix components, which affect the results [10]. Therefore, there are currently no commercial electrochemical detectors for immunochromatography. The development of new high-sensitivity signal amplifying designs could be instrumental in creating high-sensitivity immunochromatography testing systems; however, in the near future, a system of this type would likely be limited to qualitative rather than quantitative diagnostics.

## 6. Data Processing for Quantitative Immunochromatographic Assays

To proceed from the measured intensity of staining of the analyzed zone to a concentration analysis, it is necessary to use a respective calibration curve. As a rule, such calculations do not require any special efforts by the operator. The detector’s software can automatically calculate the value in question; however, finding the calibration curve’s parameters may require some preliminary measurements on standard samples with known analyte concentrations.

The procedure of quantitative assessment of immunochromatographic data includes the following stages:

(A1) obtaining calibration curves for several (typically from four to six) samples with the known concentrations of the target analyte;

(A2) choice of approximating function for calibration curves (typically linear for sandwich assay formats and sigmoidal for competitive ones);

(A3) calculation of the parameters of the asymptotic function for its best approximation to the experimental data;

(A4) conclusion about acceptability of the chosen approximation;

(A5) conclusion about range of registered signals (lines intensity) that is acceptable for obtaining quantitative assay results;

(B1) processing digital image of test strip after the assay;

(B2) identification of binding zones and calculation of marker’s binding at them;

(B3) conclusion about the correctness of the analysis by the presence of the signal from the control zone;

(B4) conclusion about the possibility of the result’s quantification by the accordance of the registered signal from the test zone to the range stated at the A5 stage;

(B5) calculation of the analyte content based on the calibration curve (see item A3);

(B6) output of measurement results and comments to them.

Stages (A1)–(A5) accord with the preliminary description of the developed methodology and stages (B1)–(B6) with the work with a specific sample. Sometimes the ratio of signals for test and control zones is used for calibration instead of the signal of the test zone.

For open detecting systems, this is obviously a precondition for the possibility of adapting a device to test systems from various manufacturers.

Considering immunochromatography as a quantitative analysis, it is important to assess the precision of its results. These parameters depend on two factors: the detector’s reproducibility of measurements and varying features in the series of test strips used in the measurements. The first factor is usually not very high, at 1%–2%, while to a substantial degree, the second parameter depends on the test system manufacturing method, the quantities of reagents applied to the analyzed zone, and the quality of the equipment, which may vary from 5% to 15%–20% for some test systems.

**Figure 5:**
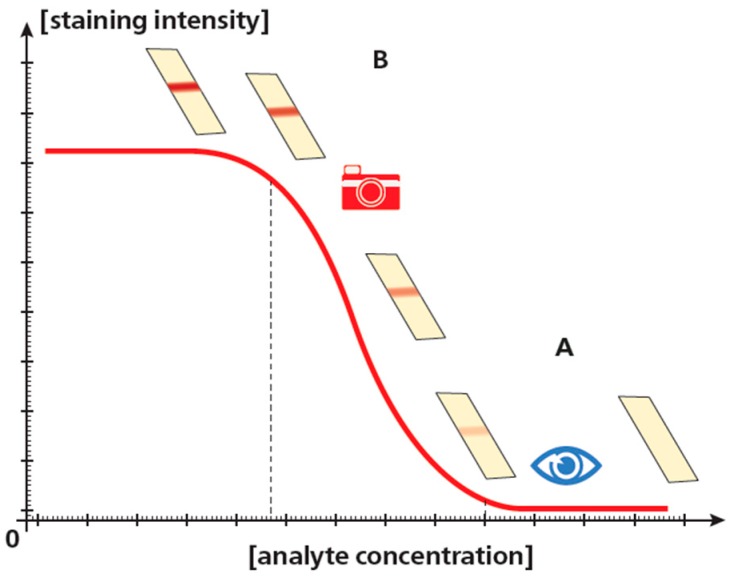
Limits of detection of the competing immunoassay (from [55], with additions and amendments). Visual detection can determine whether or not there is staining; detection limit corresponds to point A dividing these options. Instrumental detection can register staining intensity that can be compared to the values stored in the device’s memory. Detection limit corresponds to point B—the minimum concentration that causes a reliable lowering of staining intensity compared to the negative (zero) sample.

An additional important feature of quantitative processing of the assay results is the possibility of comparing different intensities of a signal instead of the comparison of its presence or absence for qualitative assessment. The reached gain is relatively small for sandwich analysis and more associated with the objectification of the conclusion. However, for competitive immunochromatography, the format’s detection limit is lowered significantly (at an order of amplitude or even more) through its shift to another sector of a concentration curve of the registered signal (Figure 5).

## 7. Advantages of Quantitation for the Results of Lateral Flow Tests

The potential for the wide use of quantitative immunochromatography is determined by the following features:

1. Detecting test results and no subjectivity in their assessments (results are explicitly assessed even for faint staining). Digital images of a test are saved for potential review of controversial results. It may serve as proof of diagnostic decisions with legal implications, such as drug tests, HIV antibody tests, and so forth.
2. Due to the analysis result’s format containing a quantitative parameter, such as the intensity of staining of a particular test strip zone, immunochromatography is considered a quantitative analysis that provides information related to the concentration of a target compound in a sample mixture.
3. Lowering the limit of detection of a compound to be detected. This gain is determined by the higher sensitivity of label detection and can become 3–50 times higher [21,56,57,58].
4. Using mobile communication devices and web-integrated devices, such as Google Glass [59], provides the following opportunities:
  - remote expert advising and supervision or even a controlled measurement process;
  - prompt integration of the transmitted test results into collected data about the patient maintained at healthcare facilities;
  - developing cloud databases using platforms developed by companies such as Mobileassay (mobileassay.com) or Fio Corporation (fio.com);
  - simple and effective statistical features of large-scale screening results.

## 8. Conclusions

Quantitative estimation of the results of immunochromatographic tests can substantially enhance the efficiency of their use in human and veterinary medicine, biosafety, consumer protection, and ecological monitoring. Actually, a row of portable detectors has been developed and implemented with the corresponding software for different kinds of immunochromatographic tests, including assays with optical, magnetic, and electrically conductive labels. While retaining the speed, cost effectiveness, and consumer friendliness of the test systems, these detectors enable detecting and processing results while precluding the subjectivity of their interpretations. New solutions based on the use of general-purpose devices such as office scanners, mobile phones, and smartphones are being developed. New, highly productive assays increase the information output of testing and allow protective actions on the obtained results to be more grounded.

## Figures and Tables

**Table 1:** Manufacturers and models of optical detectors for registering results of immunochromatography (based on [19], with actualization and additions).

Company	Country	Website	Detector Model	Mode of Measurements
Abingdon Health	United Kingdom	abingdonhealth.com	Lateral Flow Reader OEM	Colorimetry
Alere Toxicology	United Kingdom	aleretoxicology.co.uk	Alere™ DDS^®^2 Mobile Test System	Colorimetry
Axxin	Australia	axxin.com	AXXIN AX-2X	Colorimetry, fluorimetry
BD Company	United States	bd.com	BD Veritor System, BD Veritor Plus System	Colorimetry
Bio-AMD	United Kingdom	bioamd.com	Digital Strip Reader	Colorimetry
BioAssay Works	United States	bioassayworks.com	Cube-Reader	Colorimetry
Biohit Healthcare	Finland	biohithealthcare.com	Biohit Quick Test Reader (QTR)	Colorimetry
Bio-group Medical System	Italy	biogms.com	Nemesys System	Colorimetry
Charm Scientific	United States	charm.com	Charm EZ Lite System, Charm EZ System	Colorimetry
Concile	Germany	concile.de	CONCILE Ω100	Colorimetry
Detekt	United States	idetekt.com	RDS-1500, RDS-2500	Colorimetry
Dr. Fooke	Germany	fooke-labs.de	LFA Reader	Colorimetry
GenPrime	United States	genprime.com	Point-of-Care Diagnostic System	Colorimetry
Hamamatsu	Japan	hamamatsu.com	Immunochromato-Reader C10066	Colorimetry
			Immunochromato-Reader C11787	Colorimetry, fluorimetry
Hund Wetzlar	Germany	hund.de	Lateral Flow Tester LFT 100	Colorimetry
Esterline	Germany	esterline.com	cPoC Reader	Colorimetry
Maxwell Sensors	United States	maxwellsensors.com	FS-Scanner	Fluorimetry
Megalab	Greece	www.megalab.gr	Easy Reader	Colorimetry
Merck Millipore	Germany	merckmillipore.com	RQflex 20	Colorimetry
Neogen	United States	neogen.com	AccuScan GOLD	Colorimetry
Optricon	Germany	optricon.de	opTrilyzer^®^Med, opTrilyzer^®^Med Plus, Cube-Reader	Colorimetry
			opTrilyzer^®^Med Fluo	Colorimetry, fluorimetry
Qiagen	Germany	qiagen.com	ESEQuant LFR	Colorimetry, fluorimetry
Reagena	Finland	reagena.com	ReaScan	Colorimetry
Response Biomedical	Canada	responsebio.com	RAMP^®^Reader	Colorimetry, fluorimetry
Romer Labs	Austria	romerlabs.com	AgraVision™	Colorimetry
Skannex AS	Norway	skannex.com	ScanEasy, SkanFlexi X200, SkanFlexi X500	Colorimetry
Shanghai Kinbio Tech	China	kinbio.com	Kinbio DT1030n Kinbio DL2032	Colorimetry
Shenzhen Highcreation Technology	China	www.hkrgr.com	Reader HR201	Colorimetry
Sugentech	Korea	sugentech.com	INCLIX; INCLIX™ S900	Colorimetry
Synteco	Russia	synteco.ru	Reflecom Narcology, Reflecom Multitest	Colorimetry
Ushio Biomedical	Japan	www.ushiomedical.com	Point Reader V	Colorimetry
Vedalab	France	vedalab.com	Easy Reader	Colorimetry
VICAM	United States	vicam.com	Vertu Lateral Flow Reader	Colorimetry

**Table 2:** Examples of smartphone/mobile-phone-based quantitative lateral flow assays and their analytical parameters.

Detecting Device and Construction	Target Analytes	Range of Concentration Measured	Reference
UC-LFS platform	Brain natriuretic peptide	5–100 pg/mL	[34]
	Suppression of tumorigenicity 2	1–25 ng/mL	
Dual LFIA with iPhone 5s	*Salmonella enteritidis*	20–10^7^ CFU/mL	[35]
	*E. coli* O157:H7	34–10^7^ CFU/mL	
Smartphone-app-chip (SPAC) system	Aflatoxins	0.5–250 ppb	[36]
REDCap tests with iPhone	*Plasmodium falciparum*	12.5–500 parasites/μL	[37]
Smartphone Diagnostics Unit (SDU)	C-reactive protein	0.1 (amplified LFIA)/1 (nonamplified LFIA) to 100 ng/mL (for both compounds)	[38]
	Cortisol		
Smartphone’s ambient-light-sensor-based reader (SPALS-reader)	Cadmium ion	0.16–50 ng/mL	[39]
	Clenbuterol	0.046–1 ng/mL	
	Porcine epidemic diarrhea virus	0.055–20 μg/mL	
iPhone-5s-based solution	Digoxigenin	16.9–100 nM	[40]
iPhone-based solution	Cocaine	0.01–1.0 μg/mL	[41]

**Table 3:** Magnetic registering detectors for quantitative lateral flow assays.

Company	Country	Website	Detector Model	Open System
Magna BioSciences	United States	magnabiosciences.com	MICT^®^ Bench-Top System	+
Magnasense Technologies	Finland	magnasense.com	Magnasense’s Magnetometric Reader	+
VWR International	United States	vwr.com	FoodChek™’s MICT System	−

## Appendix A

**Table A1:** Examples of quantitative lateral flow tests and their working ranges reached in combination with commercial detectors.

Detector Model	Target Analytes	Range of Concentration Measured
Nemesys System (Bio-group Medical System)	Fecal occult blood	30–1000 ng/mL
	Calprotectin	20–250 µg/g
	Tetanus	0.01–0.51 IU/mL
	Celiac	8–50 IU/mL
CONCILE Ω100 (Concile)	Alpha-fetoprotein	125–200 ng/mL
	Cancer antigen 125	20–500 U/mL
	Carbohydrate antigen 15-3	10–400 U/mL
	Carbohydrate antigen 19-9	15–400 U/mL
	Carcinoembryonic antigen	2.5–150 ng/mL
	Free prostate-specific antigen	0.5–10 ng/mL
	Prostate-specific antigen	0.5–25 ng/mL
	Urinary bladder cancer antigen	5–300 µg/mL
	Human chorionic gonadotropin	5–400 mIU/mL
	Luteinizing hormone	5–200 mIU/mL
	Follicle-stimulating hormone	2.5–100 mIU/mL
	Thyroid-stimulating hormone	0.3–40 mIU/L
	Testosterone	0.25–16 ng/mL
	25-OH-vitamin D	3–100 ng/mL
	C-reactive protein	0.5–100 mg/L
	Neopterin	2.5–100 nmol/L
	Brain natriuretic peptide	100–3000 pg/mL
	Troponin I	0.5–50 ng/mL
	Heart-type fatty-acid-binding protein	2.5–100 ng/mL
	Phospholipase A2-IIA	20–500 ng/mL
Easy Reader (Vedalab)	Alpha-fetoprotein	10–300 ng/mL
	Beta-chain-specific human chorionic gonadotrophin	5–1000 IU/L
	C-peptide	0.5–40 ng/mL
	CA 125 (ovarian cancer antigen)	15–750 U/mL
	Cancer antigen 15-3	5–200 IU/mL
	Calprotectin	25–350 µg/g
	Carcinoembryonic antigen	5–250 ng/mL
	Creatine kinase MB	5–200 ng/mL
	Cortisol	25–250 ng/mL
	C-reactive protein	2.5–400 µg/mL
	High-sensitivity C-reactive protein	0.1–400 µg/mL
	Cystatin C	0.1–8 mg/L
	D-dimer	250–5000 ng/mL
	Heart-type fatty-acid-binding protein	2–120 ng/m
	Ferritin	10–630 ng/mL
	Fecal occult blood (hemoglobin)	10–499 ng/mL
	Follicle-stimulating hormone	5–400 IU/L
	Hemoglobin total	5–25 g/dL
	Glycated hemoglobin	4%–16%
	Human chorionic gonadotrophin	5–1000 IU/L
	High-sensitivity insulin	2–200 µIU/mL
	High-sensitivity prolactin	3–100 ng/mL
	High-sensitivity thyroid-stimulating hormone	1–80 mIU/L
	Immunoglobulin E	10–800 IU/mL
	Insulin	5–200 µIU/mL
	Luteinizing hormone	5–400 mIU/mL
	Microalbumin	2.5–5000 µg/mL
	Myoglobin	50–500 ng/mL
	Procalcitonin	0.3–100 ng/mL
	Prolactin	20–350 ng/mL
	Prostate-specific antigen	1–100 ng/mL
	Total T3	0.3–6 ng/mL
	Total T4	0.6–15 µg/dL
	Transferrin	4–300 ng/mL
	Troponin I	To 50 ng/mL
	Thyroid-stimulating hormone	2–80 mIU/L
	Ultrasensitive thyroid-stimulating hormone	0.2–50 mIU/L
INCLIX (Sugentech)	Thyroid-stimulating hormone, vitamin D, D-dimer	No data published
Easy Reader (Megalab)	Alpha-fetoprotein, Cancer antigen 125, Carcinoembryonic antigen, Creatine kinase MB, C-reactive protein, high-sensitivity C-reactive protein, D-Dimer, Ferritin, Fecal occult blood, Follicle-stimulating hormone, Luteinizing hormone, IgE, Insulin, Luteinizing hormone, Microalbumin, Myoglobin, Prolactin, Prostate-specific antigen, Troponin I, Thyroid-stimulating hormone	No data published
